# Dimethyl Sulfoxide Perturbs Cell Cycle Progression and Spindle Organization in Porcine Meiotic Oocytes

**DOI:** 10.1371/journal.pone.0158074

**Published:** 2016-06-27

**Authors:** Xuan Li, Yan-Kui Wang, Zhi-Qiang Song, Zhi-Qiang Du, Cai-Xia Yang

**Affiliations:** College of Animal Science and Technology, Northeast Agricultural University, Harbin, 150030, Heilongjiang, China; Inner Mongolia University, CHINA

## Abstract

Meiotic maturation of mammalian oocytes is a precisely orchestrated and complex process. Dimethyl sulfoxide (DMSO), a widely used solvent, drug, and cryoprotectant, is capable of disturbing asymmetric cytokinesis of oocyte meiosis in mice. However, in pigs, DMSO’s effect on oocyte meiosis still remains unknown. We aimed to evaluate if DMSO treatment will affect porcine oocyte meiosis and the underlying molecular changes as well. Interestingly, we did not observe the formation of the large first polar body and symmetric division for porcine oocytes treated with DMSO, contrary to findings reported in mice. 3% DMSO treatment could inhibit cumulus expansion, increase nuclear abnormality, disturb spindle organization, decrease reactive oxygen species level, and elevate mitochondrial membrane potential of porcine oocytes. There was no effect on germinal vesicle breakdown rate regardless of DMSO concentration. 3% DMSO treatment did not affect expression of genes involved in spindle organization (*Bub1 and Mad2*) and apoptosis (*NF-κB*, *Pten*, *Bcl2*, *Caspase3* and *Caspase9*), however, it significantly decreased expression levels of pluripotency genes (*Oct4*, *Sox2* and *Lin28*) in mature oocytes. Therefore, we demonstrated that disturbed cumulus expansion, chromosome alignment, spindle organization and pluripotency gene expression could be responsible for DMSO-induced porcine oocyte meiotic arrest and the lower capacity of subsequent embryo development. Our results provide new insights on DMSO’s effect on porcine oocyte meiosis and raise safety concerns over DMSO’s usage on female reproduction in both farm animals and humans.

## Introduction

Meiotic maturation of mammalian oocytes is a complex process precisely orchestrated by a wide array of endocrinal and molecular cues [1]. Upon the resumption of meiosis, oocyte undergoes a series of maturation events, from germinal vesicle breakdown (GVBD) [2], chromatin condensation into chromosomes, extrusion of the first polar body, to meiotic arrest at the second metaphase (MII) stage [3], preparing for sperm fertilization (or parthenogenetic stimulation), reactivation of the zygotic genome, and onset of early embryonic development [4]. The successful completion of meiosis and embryo development depends on a number of important factors, for instance, the correct chromosome alignment and segregation via the bipolar spindle [5], which is formed by microtubules, a cytoskeletal structure important for the extrusion of polar bodies. Thus, when aberrant spindles are present, aneuploidy will occur, devastating to oocyte meiosis and embryo development [6]. The extrusion of the first polar body also indicates the status of nuclear maturation, while the reactive oxygen species (ROS) level, activity and distribution of ooplasmic mitochondria reflect the quality of cytoplasmic maturation [7]. If both nuclear and cytoplasmic maturation are well coordinated and synchronized, oocyte will be of better quality, and embryos subsequently formed will also have better developmental competence.

Dimethyl sulfoxide (DMSO) is an amphipathic molecule, widely used as solvent to solubilize a variety of chemical substances, as cryoprotectant to vitrify cells, tissues and oocytes [8,9], and as vehicle to deliver drugs in human medicine [10]. Although low toxicity at a concentration of <10% is commonly assumed, DMSO could cause side effects when used to treat disease at different concentrations [11,12], and was found to be toxic to different types of cells (e.g. odontoblasts, colon tumor cells, astrocytes, stem cells) [13–16]. Multiple mechanisms could be possibly involved, including increase of membrane permeability, water diffusion, decrease of intracellular lipid droplets, activation of apoptosis pathway, mitochondrial damage, and change of gene expression [14,17,18].

In mice, DMSO’s effects on oocytes and embryos could be summarized together, including extrusion of a large first polar body during meiosis [19], inhibition of spontaneous fragmentation during *in vitro* oocyte aging [20] and promotion of the somatic cell nuclei reprogramming for reconstructed embryos [21]. As a non-rodent mammalian system, porcine oocyte has cytoskeletal regulatory mechanisms similar to humans but different from mice [22]. Previous studies have used this non-rodent system to investigate the effects of Griseofulvin [22] and Aflatoxin B1 [23] on oocyte maturation.

However, DMSO’s effect on porcine oocyte meiosis is still unclear. Therefore, in the present study, we *in vitro* matured porcine cumulus-oocyte complexes (COCs) with DMSO supplementation, to observe the cumulus expansion, cell cycle progression and nuclear status of oocytes; to detect the ROS level, mitochondrial membrane potential (ΔΨm) and spindle organization in mature oocytes; to investigate the expression of genes involved in pluripotency, spindle organization and apoptosis signaling pathways; and finally, to assess the developmental capacity of DMSO treated MII oocytes. Interestingly, contrary to what was found in mice, we did not observe the formation of the large first polar body and symmetric division in porcine oocytes treated with 0.1% to 4% DMSO. Our results provided new insights on DMSO’s usage in assisted reproductive technology for both farm animals and humans.

## Materials and Methods

### Ethics statement

All experimental materials and procedures taken in this study were reviewed and approved by the Animal Care Commission and Ethics Committee of Northeast Agricultural University.

### Chemicals

All reagents were purchased from Sigma (St. Louis, MO, USA), unless otherwise stated. Antibodies for immunofluorescence were purchased from ABclonal (Nanjing, China). DMSO (Sigma, cat. no. D2650) suitable for cell culture was used in the present study.

### Collection and *in vitro* maturation (IVM) of pig cumulus-oocyte complexes (COCs)

Porcine ovaries were collected from a local slaughterhouse and transported to the laboratory while maintained at 30–35°C. Follicular fluids were aspirated from antral follicles (about 3-6mm in diameter) using an 18-gauge needle attached to a 10ml disposable syringe, and washed three times in TL-HEPES-PVA (114mM NaCl, 3.2mM KCl, 2mM CaCl_2_·2H_2_O, 0.34mM Na_2_HPO_4_, 0.5mM MgCl_2_, 10mM Na Lactate, 10mM HEPES, 0.2mM Na Pyruvate, 12mM Sorbitol, 2mM NaHCO_3_, 0.1mg/ml polyvinylalcohol (PVA), 1μg/ml gentamicin) [24]. Then COCs were picked up, those with more than three layers of cumulus cells and uniform ooplasm were selected, and washed three times in maturation medium (TCM 199 medium (Gibco BRL, Grand Island, NY) supplemented with 0.1% PVA, 3.05mM D-glucose, 0.91mM sodium pyruvate, 1μg/ml gentamicin, 0.57mM cysteine, 0.5μg/ml luteinizing hormone, 0.5μg/ml follicle stimulating hormone, 10ng/ml epidermal growth factor). Then approximately 50 COCs were transferred into 500μl maturation medium covered with mineral oil in a 24-well plate, and cultured in an incubator (39°C, 5% CO_2_, and saturated humid air) for 44h. Degenerated oocytes with few or devoid of cumulus cells were discarded. Porcine COCs were *in vitro* cultured in maturation medium supplemented with DMSO to reach a final concentration (v/v) as desired (0%, 0.1%, 1%, 2%, 3% and 4%).

### Evaluation of cumulus expansion and oocyte nuclear status

Cumulus expansion of COCs was assessed at 0, 24 and 44h of IVM as described previously [25]. At each time point, a digital image of each COC was captured using the same magnification and parameters under an inverted microscope (Olympus, Tokyo, Japan). The total two-dimensional area of each COC was measured and calculated using the Image J software (version 1.47v) [26]. The calculated area at 0h was treated as the reference value, and used to calculate the relative ratios of cumulus expansion at 24h and 44h.

To assess the nuclear status, the cumulus cells of COCs were separated from oocytes by gentle vortexing in 0.1% hyaluronidase in HEPES-buffered Tyrode medium containing 0.01% PVA. Denuded oocytes were observed, and images were acquired under an inverted microscope (Olympus, Tokyo, Japan). Then, oocytes were fixed in 4% paraformaldehyde in phosphate buffer solution (PBS) for 40min at room temperature and stained with 10μg/ml Hoechst33342 in PBS for 10min at RT. After a wash in PBS, oocytes were mounted onto glass slides in ProLong Diamond Antifade Mountant reagent (Life Technologies, USA), and then observed under an inverted fluorescence microscope (Olympus, Tokyo, Japan). The stages of oocyte nuclei were classified as germinal vesicle (GV), pro-metaphase I (pro-MI), metaphase I (MI), anaphase I (AI), telophase I (TI), and MII, respectively [27].

### Immunocytochemistry staining

Denuded MII oocytes were fixed in 4% paraformaldehyde in PBS for 40min at room temperature, permeabilized with 1% Triton X-100 in PBS overnight at 4°C, and then blocked with 1% bovine serum albumin (BSA) in PBS for 1h at room temperature. Samples were then incubated with rabbit anti-α-tubulin polyclonal antibody (1:50 in blocking buffer) overnight at 4°C. After three washes in PBS supplemented with 0.01% Triton X-100 and 0.1% Tween-20 (PBST, 10min each), respectively, oocytes were incubated in second antibody (FITC conjugated goat anti-rabbit IgG (H+L) 1:150 in blocking buffer) for 1h at room temperature. Then after washing three times with PBST (10min each), samples were stained with propidium iodide (10μg/ml in PBS) for 10min at room temperature. Finally, oocytes were mounted onto glass slides in ProLong Diamond Antifade Mountant reagent (Life Technologies, USA). Samples incubated without the primary or secondary antibodies were treated as negative controls. A Nikon fluorescence microscope (Nikon 80i, Japan) was used to take fluorescent images (at 100× magnification) of each oocyte to assess chromosomal alignment and spindle assembly, respectively [28]. Representative images were also taken, using a Laica laser-scanning confocal microscope (Laica, Germany).

### Measurement of reactive oxygen species (ROS) and mitochondrial membrane potential (ΔΨm)

The ROS level of denuded oocyte at MII stage was measured according to a method published previously [29]. Briefly, oocytes were incubated in 10μM 2’,7’-dichlorodihydrofluorescein diacetate (DCFH-DA) in PBS for 30min at room temperature, followed by three washes in PBS, and then put into 10μl droplets of PBS to take the fluorescent images under the inverted fluorescence microscope (Olympus, Tokyo, Japan). Each image was taken with the same parameters, then the fluorescence intensity of each oocyte was quantified using the Image J software, and the relative fluorescence intensity ratio was calculated by treating the value obtained for the control group as the reference.

The alterations of mitochondrial ΔΨm in denuded MII oocytes were assessed with the Rhodamine 123 (RH123) staining method according to a previous report with slight modifications [30]. RH123 is a fluorescent probe, which could enter and remain in the mitochondria if ΔΨm is intact, and *vice versa* [31]. Briefly, MII oocytes were incubated with RH123 (5μg/ml) for 30min at 37°C in dark, and then washed with PBS. The fluorescent images of samples were taken and analyzed in a similar way as mentioned above for ROS level.

### Parthenogenetic activation and embryo culture

Denuded MII oocytes were equilibrated in droplets of activation medium (0.28M mannitol, 0.1mM CaCl_2_·2H_2_O, 0.1mM MgCl_2_, 1mg/ml BSA, 0.5mM Hepes), and two direct current pulses of 1.2kV/cm for 30μs were applied to stimulate oocytes using Electrocell Manipulator (BTX830, USA). Then, oocytes were incubated for 4h in PZM-3 containing 2.5mM 6-dimethylaminopurine (6-DMAP) and 5μg/ml cytochalasin B (CB) in an incubator at 39°C, 5% CO_2_ and saturated humid air. Activated embryos were cultured in PZM-3 medium covered with mineral oil in the incubator (5% CO_2_ in humidified air at 39°C) for 7 days. Embryonic cleavage and blastocyst rates were evaluated at 48h and 168h post-activation, respectively [32]. The total cell number of blastocyst was calculated as previously described with slight modifications [33]. Briefly, all blastocyst were fixed in 4% paraformaldehyde in PBS for 40min, stained with 10μg/ml Hoechst33342 for 10min at room temperature, mounted onto glass slides in ProLong Diamond Antifade Mountant reagent (Life Technologies, USA), and finally examined under a fluorescence microscopy (Olympus, Tokyo, Japan) using an excitation filter of 355nm to count for the cell number.

### RNA extraction and RT-qPCR

Denuded oocytes were washed twice with PBS (without Ca^2+^ and Mg^2+^) and stored at −80°C. Total RNA was extracted from 60 MII-stage oocytes from the control group, 60 MII oocytes from 3% DMSO treated (44h) group and 60 non-MII oocytes from 3% DMSO treated (44h) group (n = 3 replicates for each group), respectively, using RNeasy Mini Kit (Qiagen, USA) according to the manufacture's instructions. RNase-free DNase was added to remove genomic DNA. Total RNA from each sample was synthesized into cDNA in a 20μl final volume of reverse transcription system using the ABI kit following the instructions (Life Technologies). Primers were designed by primer-blast, as shown in S1 Table. Quantitative PCR was conducted in a 10μl reaction volume including 1μl cDNA template, primers and Roche FastStart Universal SYBR green master mix (Roche Molecular Systems) using a 7500 real-time PCR detection system (Applied Biosystems, Carlsbad, CA, USA). Thermal cycling parameters were setup as following: 95°C for 10min, followed by 40 cycles at 95°C for 15sec and 60°C for 1min. Transcripts were quantified in triplicates for each sample, and Ywhag was used as the reference gene. Relative abundance was calculated using the comparative Ct (2^−ΔΔCt^) method [34].

### Statistical analysis

Statistical analyses were performed in SPSS19 (SPSS Inc., Chicago, IL, USA) and SAS9.1 (SAS Institute Inc., Cary, NC, USA). Each experiment was repeated at least three times. The differences in the percentages of GVBD, nuclear status, normal spindle, embryo cleavage and blastocyst were compared using Chi-square test or Fisher’s exact test followed by multiple comparisons with Bonferroni correction (SPSS19). One-way ANOVA was used to analyze the data of cell number per blastocyst and Student’s T-test was used to compare the differences in ROS and mitochondrial ΔΨm levels (SPSS19). Differences in cumulus expansion and gene expression were analyzed using the linear mixed model procedure (SAS9.1). The statistical differences were determined to be significant when p < 0.05 or the most significant when p < 0.01. Data are displayed as mean±standard error of the mean (SEM).

## Results

### DMSO inhibited cumulus expansion and affected nuclear maturation

**Fig 1:**
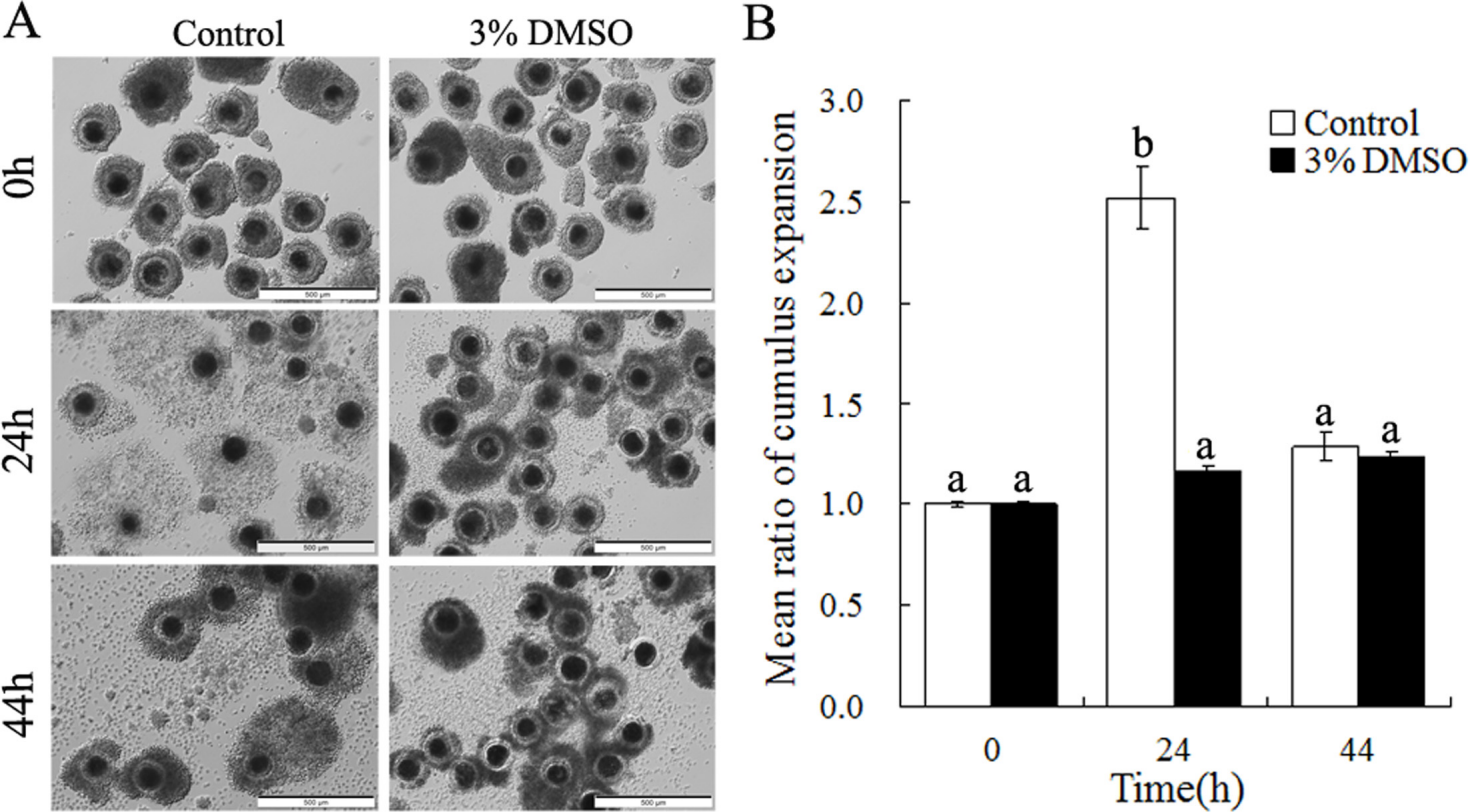
Impact of DMSO on cumulus expansion of COCs during IVM. (A) Representative images of COCs taken at three time points (0, 24 and 44h) during IVM. Scale bar, 500μm. (B) The extent of cumulus expansion of COCs was presented as a ratio of the total two-dimensional area of each COCs at different time points to that of the original COCs at 0h. Three replicate experiments were performed. Different lower case superscripts (a-b) indicate significant differences (p < 0.05).

Generally, for the control group, we observed cumulus cells first expanding at 24h and then contracting at 44h, comparing to the starting point at 0h (relative indices, 2.52 at 24h, 1.28 at 44h versus 1.0 at 0h, Fig 1). However, by adding DMSO into the maturation media (at different final concentrations, 0.1%, 1%, 2%, 3% or 4%) to culture porcine COCs, we observed accordingly a dose-dependent suppression effect of DMSO on cumulus expansion (S1 Fig). In addition, when we analyzed the 3% DMSO treatment on cumulus expansion in detail, relative indices of cumulus expansion were found to be without significant changes during the entire process of IVM (1.16 at 24h, 1.22 at 44h versus 1.0 at 0h, Fig 1, p > 0.05), showing different change dynamics as compared to the control group. When further compared the two groups at different time points, we found that cumulus expansion at 24h of IVM in 3% DMSO treatment group (n = 150) was significantly inhibited (1.16 versus 2.52) (p < 0.05), while there were not different at 44h of IVM (1.28 and 1.22, p > 0.05).

**Fig 2:**
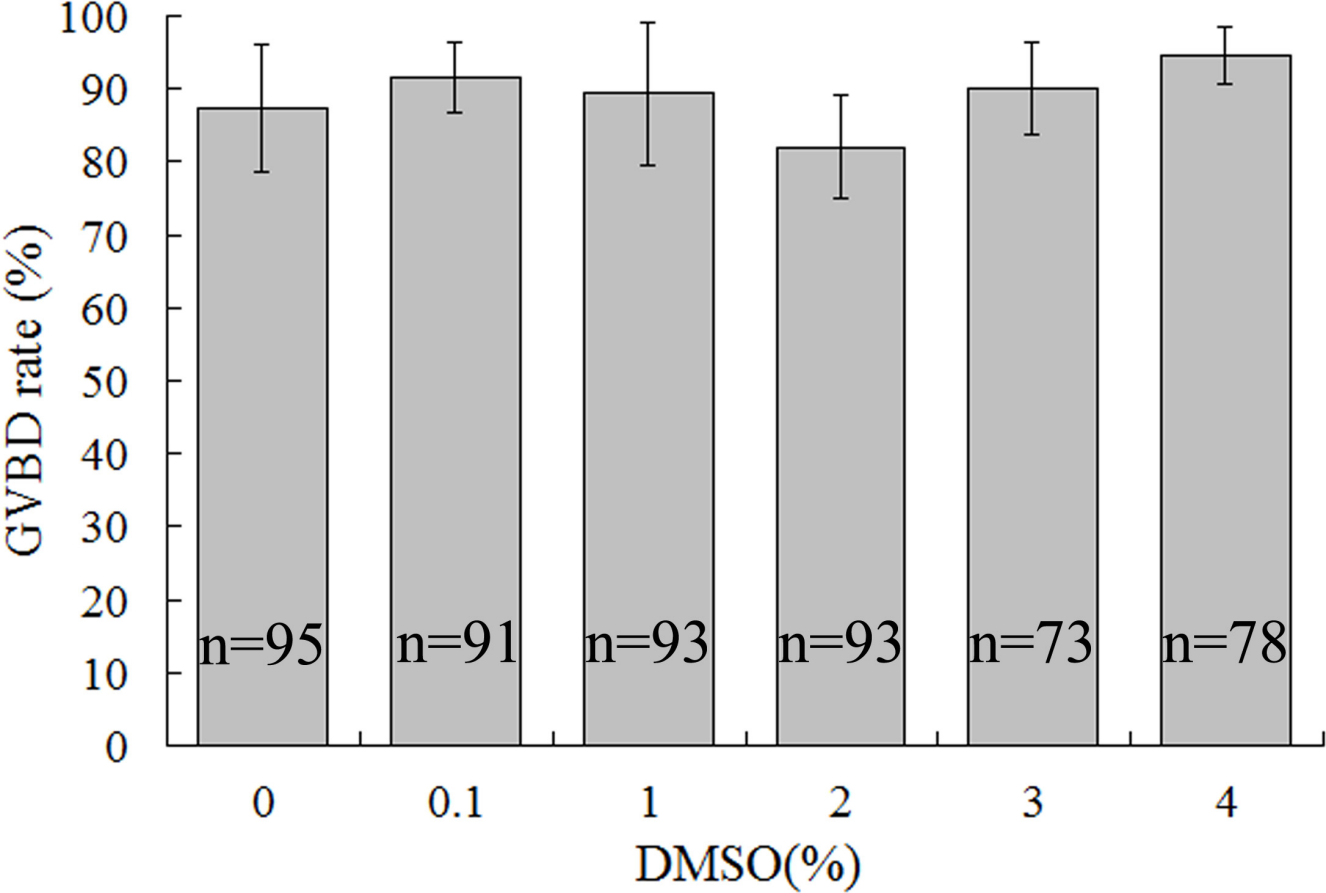
Effects of DMSO treatment on rates of oocytes with germinal vesicle breakdown. COCs were treated with 0%, 0.1%, 1%, 2%, 3%, or 4% DMSO for 24h, and then GVBD rates of oocytes were evaluated by Hoechst33342 staining.

We further examined DMSO’s effect on oocyte meiotic resumption in COCs, indicated by GVBD rates at 24h of IVM. However, GVBD rates were not different between the control and DMSO treated groups regardless of concentrations (p > 0.05), indicating that DMSO had no effect on meiotic resumption (Fig 2).

**Fig 3:**
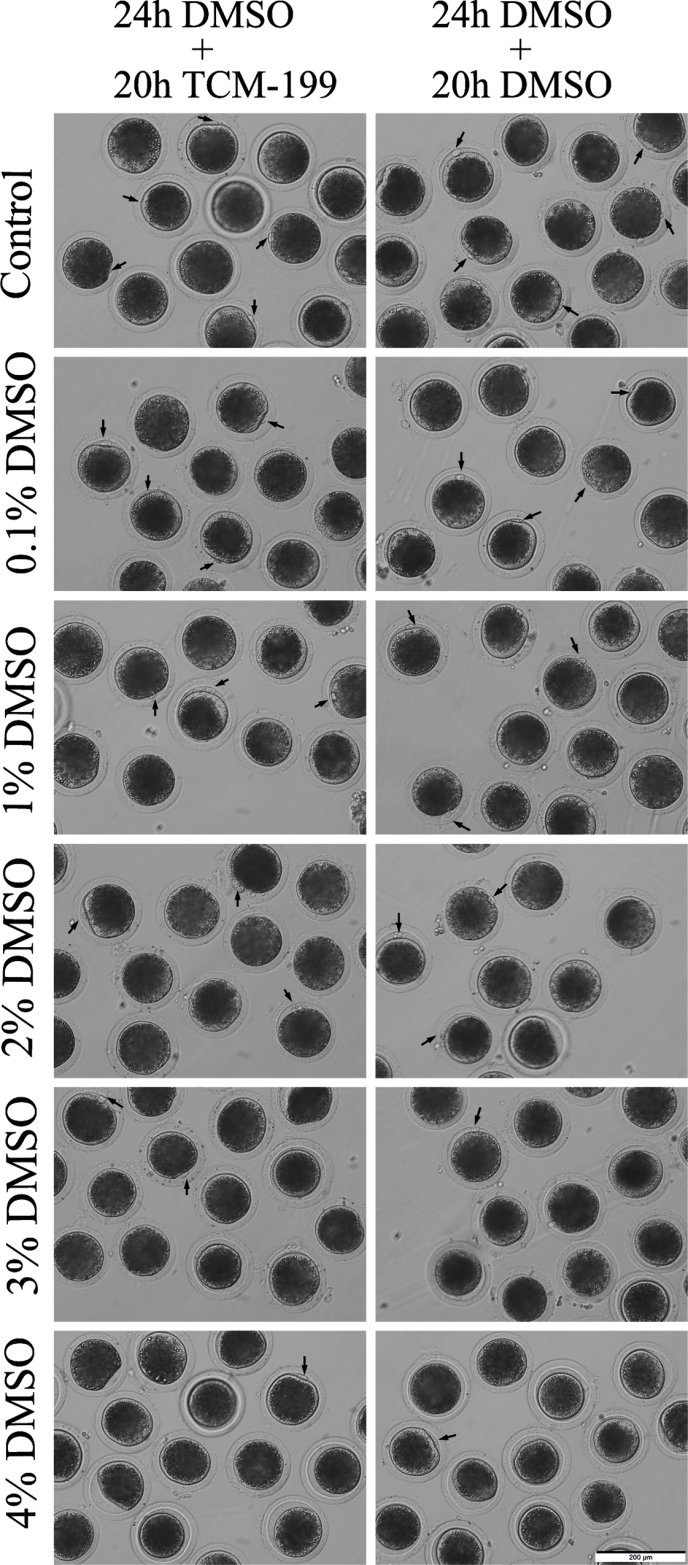
Morphology of oocytes after DMSO treatment. COCs were cultured *in vitro* with maturation medium supplemented with 0%, 0.1%, 1%, 2%, 3% and 4% DMSO for 24h, and then transferred into without (termed as the 24h DMSO+20h TCM-199 group) or with DMSO (termed as the 24h DMSO+20h DMSO group) maturation medium to mature for 20h. Then cumulus cells were stripped off to take images for denuded oocytes. Arrows indicated the normal sized first polar bodies of MII oocytes. Scale bar, 200μm.

Next, we assayed DMSO’s effect on nuclear maturation. Following DMSO treatment for the first 24h of IVM, COCs were monitored continuously for the remaining 20h of IVM period without (termed as 24h DMSO+20h TCM-199 treatment group) or with (termed as 24h DMSO+20h DMSO treatment group) DMSO supplementation. After stripping of cumulus cells at 44h of IVM, the oocytes from both groups were observed under an inverted microscope and we did not find any oocytes with the larger first polar body (Fig 3) as reported in mice [19]. Then denuded oocytes were stained to observe which stage of cell cycle oocyte nuclei were at. Overall, we found DMSO could inhibit nuclear maturation in a dose- and time-dependent manner for both types of DMSO treatments (24h DMSO+20h TCM-199 treatment group and 24h DMSO+20h DMSO treatment group), and high-dose DMSO exposure for a relatively longer time could result in meiotic arrest partially through inducing chromatin abnormality. In the 24h DMSO+20h TCM-199 treatment group, 4% DMSO treatment significantly decreased the maturation rate (indicated as the rate of first polar body extrusion) as compared to the control (30.0% versus 69.7%) and other groups (p < 0.05, Table 1), through significantly increasing the rate of abnormal nuclei (26.3% versus 2.3%) (p < 0.05, Table 1, S2 Fig and S2 Table), suggesting that treatment of COCs by 4% DMSO for the first 24h affected the subsequent nuclear maturation following meiotic resumption.

**Table 1:** Nuclear status of porcine oocytes from COCs *in vitro* matured with DMSO for the first 24h and then for 20h with TCM-199 (24h DMSO+20h TCM-199 group).

DMSO (%)	No. oocytes	No. GV (%±SEM)	No. proMI (%±SEM)	No. MI (%±SEM)	No. AI+TI (%±SEM)	No. MII (%±SEM)	No. Abnor. (%±SEM)
0	109	1(0.7±0.8)^a^	3(2.9±2.5)^a^	22(21.0±4.6)^a^	3(3.3±2.5)^a^	77(69.7±4.0)^a^	3(2.3±1.6)^a^
0.1	110	1(1.1±1.2)^a^	0(0.0±0.0)^a^	24(21.7±2.1)^a^	4(4.0±3.6)^a^	77(69.7±4.0)^a^	4(3.5±1.5)^ab^
1	113	0(0.0±0.0)^a^	1(0.8±1.0)^a^	38(32.1±7.7)^a^	7(5.8±2.6)^a^	63(58.1±11.3)^a^	4(3.1±2.1)^a^
2	102	2(2.0±1.3)^a^	0(0.0±0.0)^a^	28(26.6±4.7)^a^	7(6.6±2.8)^a^	60(59.5±3.7)^a^	5(5.3±3.6)^ab^
3	99	2(2.0±1.4)^a^	0(0.0±0.0)^a^	24(23.8±2.7)^a^	4(4.1±2.3)^a^	54(55.3±3.7)^a^	15(14.8±3.9)^bc^
4	107	4(3.8±3.1)^a^	0(0.0±0.0)^a^	31(28.8±7.9)^a^	12(11.1±2.6)^a^	33(30.0±9.4)^b^	27(26.3±8.4)^c^

Note: Within the same column, different superscripts indicate significant differences (p < 0.05). Abbreviations: GV, germinal vesicle; proMI, prometaphase I; MI, metaphase I; AI+TI, anaphase I and telophase I; MII, metaphase II; Abnor., nuclear abnormality.

In contrast, in the 24h DMSO+20h DMSO treatment group, 3% DMSO already significantly decreased the maturation rate as compared to the control group (35.2% versus 72.4%, p < 0.05, Table 2), via inducing more oocytes arresting at AI+TI stages (23.3% in 3% DMSO treatment group versus 4.2% in the control group). Similarly, further significant decrease of maturation rate was observed for 4% DMSO treatment group (9.5%, p < 0.05, Table 2), possibly through increasing the rate of oocytes with nuclear abnormality (42.0%) (observed as induced chromatin condensation or fragmentation) (S2 Fig and S2 Table).

**Table 2:** Nuclear status of porcine oocytes from COCs *in vitro* matured with DMSO for 44h (24h DMSO + 20h DMSO group).

DMSO(%)	No. oocyte	No.GV (%±SEM)	No.proMI (%±SEM)	No.MI (%±SEM)	No.AI+TI (%±SEM)	No.MII (%±SEM)	No.Abnor. (%±SEM)
0	94	0(0±0)^a^	2(2.0±1.2)^a^	18(19.4±2.9)^a^	4(4.2±1.1)^a^	68(72.4±1.7)^ab^	2(2.0±1.2)^a^
0.1	97	1(1.0±1.2)^a^	0(0±0)^a^	14(14.6±5.6)^a^	4(4.2±2.6)^a^	77(79.2±7.8)^a^	1(1.0±1.2)^a^
1	82	4(4.9±3.8)^a^	2(2.5±1.6)^a^	18(22.1±5.0)^a^	3(3.5±2.5)^a^	54(65.8±2.0)^ab^	1(1.1±1.4)^a^
2	89	2(2.2±1.4)^a^	0(0±0)^a^	27(29.6±8.2)^a^	7(8.1±1.9)^ab^	49(56.1±9.6)^bc^	4(4.1±3.1)^a^
3	94	4(4.2±2.6)^a^	2(2.1±1.3)^a^	26(27.6±3.4)^a^	22(23.3±6.9)^b^	33(35.2±6.4)^c^	7(7.5±2.7)^a^
4	81	4(4.8±0.9)^a^	6(7.1±1.8)^a^	21(25.2±5.0)^a^	10(11.4±7.7)^ab^	8(9.5±6.8)^d^	32(42.0±19.6)^b^

Note: Within the same column, numbers with different superscripts differ significantly (p < 0.05).

### DMSO decreased ROS production and increased mitochondrial ΔΨm

**Fig 4:**
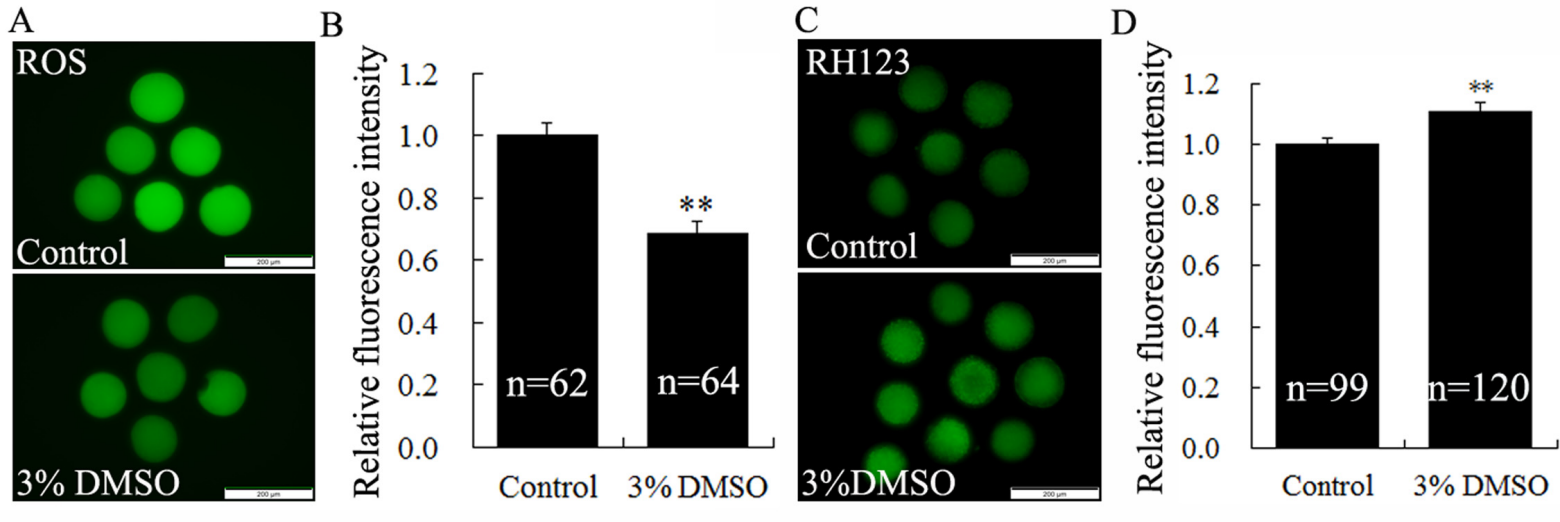
Effects of DMSO treatment on the ROS level and mitochondrial membrane potential of *in vitro* matured porcine oocytes. Representative fluorescence images were taken from porcine mature oocytes stained with DCFH-DA (A) and RH123 (C). MII oocytes were derived from the control and 3% DMSO treated (44h) COC groups, respectively. The graphs were presented as relative fluorescence intensity of ROS (B) and RH123 (D) in pig MII oocytes. * indicates significant difference at p < 0.05 level, and ** at p < 0.01 level. Scale bar, 200μm.

In order to assess DMSO’s effect on ooplasmic quality, we examined the ROS level and mitochondrial ΔΨm of MII oocytes treated by 3% DMSO (Fig 4). The relative fluorescence level of ROS was significantly reduced in the 3% DMSO group as compared to the control group (Fig 4A and 4B, p < 0.01), while the relative RH123 fluorescence level increased significantly (Fig 4C and 4D, p < 0.01), which suggests that 3% DMSO treatment could accelerate cytoplasmic maturation process of porcine mature oocytes possibly through alleviating ROS level but elevating mitochondrial ΔΨm.

### DMSO treatment impaired chromosomal alignment and spindle organization

**Fig 5:**
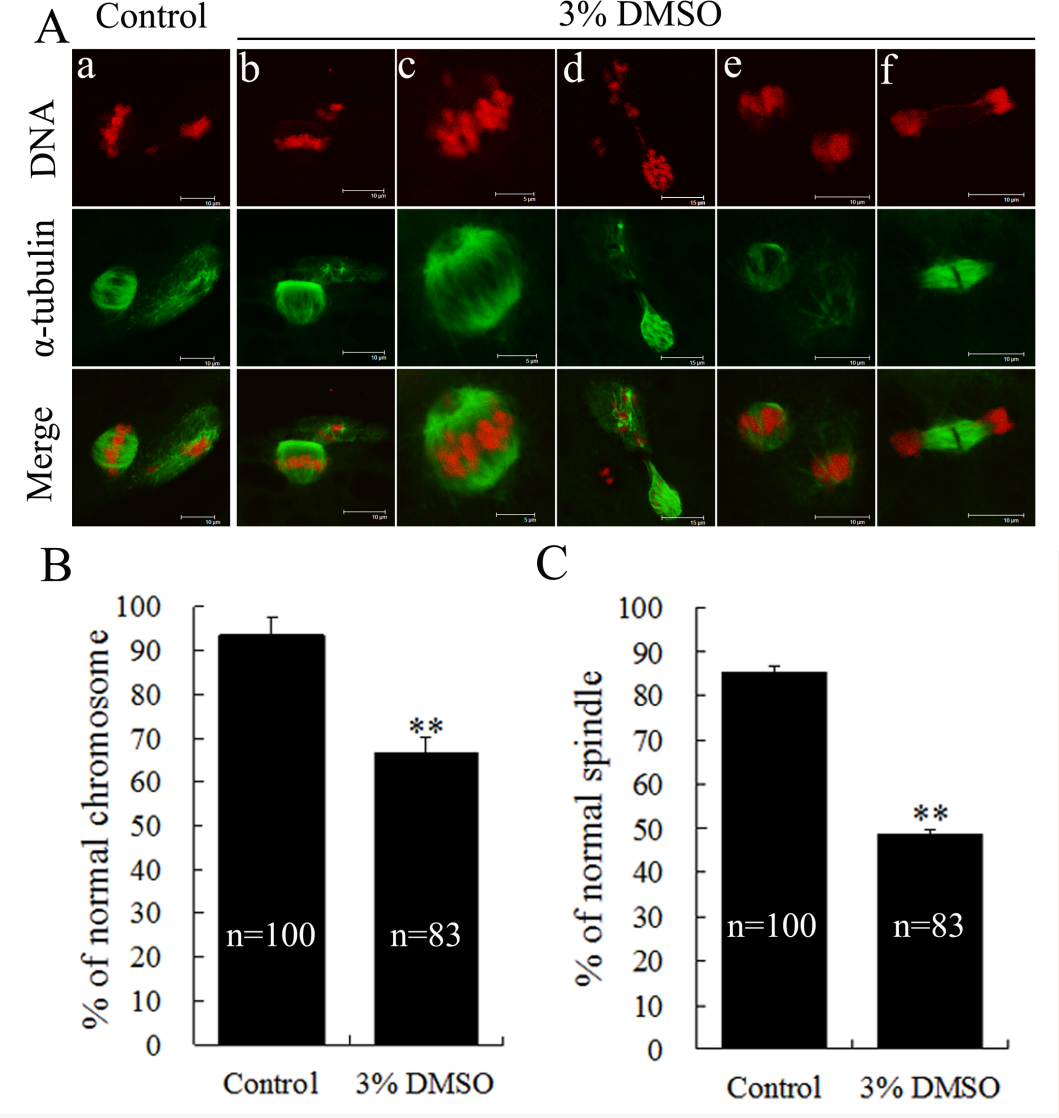
Effects of DMSO on chromosome alignment and spindle organization of *in vitro* matured porcine oocytes. (A) Representative fluorescence images of porcine mature oocytes showed spindle morphology (green) and chromosome alignment (red). Control oocytes displayed bipolar barrel-shaped spindles and well-aligned chromosomes on the metaphase equator (a), whereas chromosome misalignment and spindle defects were frequently observed in 3% DMSO treatment group, which can be classified into five different types as described in the results section (b-f). Percentages of MII oocytes with normal chromosome alignment (B) and spindle organization (C) were presented as mean ± SEM from three independent experiments. ** indicates the most significant difference (p < 0.01).

For the control group, chromosomes of most MII oocytes were found to be well-aligned at the metaphase equator, and the bipolar-shaped spindle organization could also be seen (Fig 5A-a). However, MII oocytes treated by 3% DMSO for 44h were classified into five groups according to chromosome misalignment and/or spindle defects, including 1) normal chromosomes, and the monopolar spindle (Fig 5A-b); 2) normal spindle, but chromosomes dislocated from the metaphase plate (Fig 5A-c); 3) some chromosomes displaced from the metaphase plate, and abnormal spindle (Fig 5A-d); 4) chromosomes highly condensed, and the spindle almost completely disassembled (Fig 5A-e); and 5) chromosomes and spindle both severely disrupted (Fig 5A-f). Further analysis showed that in the 3% DMSO treatment group rather than the control group, mature oocytes were of significantly lower percentage with regard to normal chromosome alignment (67% versus 93%, p < 0.01, Fig 5B) and normally shaped spindles (48% versus 85%, p < 0.01, Fig 5C), suggesting that high-dose DMSO exposure during meiotic maturation could induce chromosomal abnormality and spindle defects of mature oocytes.

### DMSO exposure affected subsequent embryonic development of MII oocytes

Next, we evaluated if DMSO treatment could affect the subsequent developmental capacity of mature MII oocytes following parthenogenetic stimulation. Significant decreases were observed for embryonic cleavage and blastocyst rates of parthenotes in 3% DMSO treatment group as compared to 1% DMSO treatment group and the control group (cleavage rates, 62.1%, 81.2% and 83.2%; blastocyst rates, 4.5%, 16.3% and 27.6%, respectively) (p < 0.05, Table 3), while no differences were found for cell numbers of blastocyst among three groups (p > 0.05, Table 3), suggesting that 3% DMSO exposure during IVM could affect the subsequent embryonic development.

**Table 3:** Development of parthenotes derived from mature oocytes following DMSO treatment for 44h during IVM.

DMSO(%)	No. embryos	No.≧2-cell embryos (%±SEM)	No. blastocysts (%±SEM)	Cell number per blastocyst
0	119	101(83.2±6.2)^a^	32(27.6±8.6)^a^	40.0±1.3^a^
1	148	120(81.2±1.6)^a^	25(16.3±5.7)^a^	38.0±1.5^a^
3	156	97(62.1±5.1)^b^	7(4.5±3.1)^b^	37.5±9.9^a^

Note: Within the same column, different superscripts mean significant differences (p < 0.05).

### DMSO unaltered the expression of genes involved in spindle organization and apoptosis, but decreased the pluripotency gene expression

**Fig 6:**
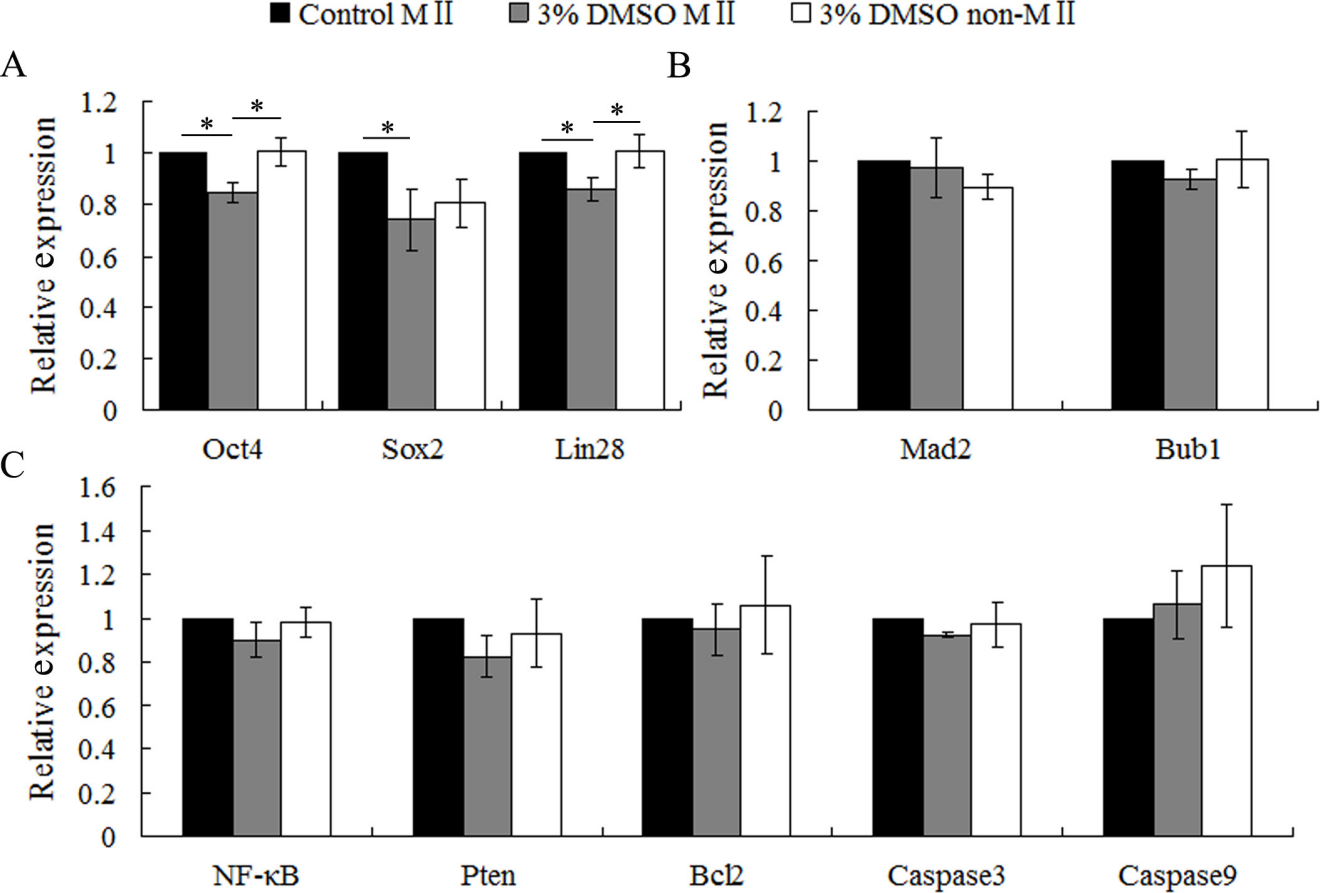
Effect of DMSO treatment on gene expression levels. MII-stage oocytes from the control group, MII oocytes from 3% DMSO treated (44h) group and non-MII oocytes from 3% DMSO treated (44h) group were respectively collected for RT-qPCR analysis as described in the section of Materials and Methods. 3% DMSO treatment changed mRNA expression levels of pluripotency genes (*Sox2*, *Oct4* and *Lin28*) (p < 0.05) (A), but not spindle organization related genes (*Mad2* and *Bub1*) (B) and apoptosis genes (*NF-κB*, *Pten*, *Bcl2*, *Caspase3* and *Caspase9*) (C) (p > 0.05) in MII oocytes, when compared to the control group.

We further investigated the role of DMSO treatment during oocyte meiosis on expression levels of genes involved in pluripotency, spindle organization and apoptosis. RT-qPCR results revealed that 3% DMSO treatment significantly decreased the relative abundance of pluripotency genes (*Oct4*, *Sox2*, and *Lin2*, p < 0.05), but not spindle organization (*Bub1* and *Mad2*) and apoptosis related genes (*NF-κB*, *Pten*, *Bcl2*, *Caspase3* and *Caspase9*) (p > 0.05) in MII oocytes when compared to the control group (Fig 6). Therefore, DMSO might cause subsequent developmental failure through affecting genes involved in the pluripotency pathway.

## Discussion

In the current study, we demonstrated that DMSO could have multiple adverse effects on the maturation of porcine COCs. Since DMSO is frequently and directly used not only in human medicine and as research reagent, but also widely as cryoprotectant (usually 10% DMSO) to cryo-preserve biological materials, such as swine ovarian tissue (10% DMSO) [35], immature and mature porcine oocytes (17.5–35% DMSO) [36], it is highly probable that human and animal tissues, as well as their gametes could be exposed to high doses of DMSO. Therefore, our observation that DMSO has adverse effects on oocyte maturation necessitates and warrants further detailed investigation on DMSO’s effects on other related areas of assisted female reproduction.

To obtain oocytes with good quality and ensure a better outcome of subsequent embryonic development, cumulus cells are inevitable by establishing bidirectional communication with oocyte cargo [37]. Pig COC system has been previously used to investigate the effects of Griseofulvin [22] and Aflatoxin B1 [23] on oocyte maturation. By using the IVM system of COCs, in the present study we showed that DMSO influenced cumulus expansion in a concentration-dependent manner, which may act through the activation of apoptosis pathway in cumulus cells. A previous report supported that DMSO induces rat retinal apoptosis *in vivo* and rat retinal neuronal cell apoptosis *in vitro* [12]. Thus, the inhibition of cumulus expansion of COCs by DMSO could be one possible reason for the poor developmental capacity of parthenotes, which is in line with previous findings that porcine oocytes without surrounding cumulus cells during IVM have significantly bad capacity regarding subsequent parthenote development [37,38]. Further experiments are needed to investigate what aspects of cumulus cells affected by DMSO treatment and their influence on the ability of oocyte maturation and embryo development.

In addition, we observed that DMSO induced meiotic cycle arrest in a time- and dose-dependent manner through disturbing chromosomal alignment and condensation of chromatin/chromosomes, which possibly due to that DMSO can release Ca^2+^ from intracellular stores [39,40]. As reported previously, 3% DMSO treatment can induce a high proportion of mouse MII oocytes to be abnormal with larger first polar bodies, by changing the organization of spindle and actin, and then disrupting cytokinesis completion [19]. In the present study, we found that 3% DMSO treatment for 44h during porcine oocyte IVM significantly decreased the maturation rate, and markedly increased the abnormality of chromosome alignment and spindle organization of MII oocytes, whereas no MII oocytes with the larger first polar body were observed, indicating that DMSO did not affect the asymmetric cytokinesis of porcine oocytes. The different effects of DMSO on porcine and rodent oocyte meiosis likely come from the underlying differences in meiotic spindle dynamic organization and centrosomal composition. In non-rodent species like swine, the meiotic spindle is absent of cytoplasmic centrosomes and asters, and the major centrosomal composition is the nuclear mitotic apparatus protein NuMA, while in mice, the meiotic spindle is formed by cytoplasmic centrosomal foci aggregated into asters that further migrate and form spindle, and the major component of mouse centrosomes isγ-tubulin [22,41,42]. In addition, another previous study has shown that DMSO does not affect cytokinesis of activated mouse oocytes, suggesting that DMSO’s effects on mouse oocyte cytokinesis relate to the activation status [20]. Furthermore, 1% DMSO included in activation medium has been shown to significantly improve the blastocyst and full-term developmental rates of mouse cumulus-oocyte reconstructed pairs, indicating that DMSO might affect reprogramming of somatic cell nuclei after or during oocyte activation [21]. In porcine SCNT, whether DMSO has a similar effect is still unknown.

In humans, DMSO has been shown to be able to down-regulate mRNA and protein expression of pluripotency genes of *Oct4*, *Sox2* and *Nanog* in embryonic stem cells [17, 18] and embryonic carcinomas cells [43], and up-regulate the protein expression of tumor suppressor gene *Pten* through activation of *NF-κB* in promyelocytic leukemia cells [44]. In rat retinal neuronal cells, 2–4% DMSO has been found to induce cell death through *Caspase3*-independent pathway [11]. In the current study, we also found that mRNA expression levels of genes involved in pluripotency (*Oct4*, *Sox2* and *Lin28*) were down-regulated significantly in MII oocyte, but not in arrest oocytes by 3% DMSO treatment. However, apoptosis (*NF-κB*, *Pten*, *Bcl2*, *Caspase3* and *Caspase9*) pathways showed no differences between DMSO-treated and the control porcine oocytes. Therefore, DMSO could directly affect pluripotency-related developmental programs for humans and pigs, however, the underlying molecular mechanism needs further investigation. Moreover, although spindle defects were observed in DMSO treated porcine MII oocytes, mRNA levels of *Mad2* and *Bub1* were unchanged. Furthermore, it has been shown that 5% DMSO treatment of cultured mouse astrocytes for 24h significantly increased ROS level, and decreased mitochondrial ΔΨm [14], which is different from our findings in the present study, also suggesting that different systems might have species-specific drug sensitivity and response pathways.

We further demonstrated that porcine parthenotes derived from MII oocytes following 3% DMSO treatment exhibited significantly lower cleavage and blastocyste rates, although the ROS level was lower and mitochondrial ΔΨm was higher as compared to those in the control group. Major reasons for the poor embryo development caused by 3% DMSO treatment likely come from chromosomal abnormality, spindle defects, pluripotency gene expression decrease and the discrepancy between ooplasmic and nuclear maturation status [7,45].

In summary, the present study reveals that disturbed cumulus expansion, chromosome alignment, spindle organization and pluripotency gene expression could be potentially involved in DMSO-induced porcine oocyte meiotic arrest and the lower capacity of subsequent embryo development. Our data obtained using a non-rodent porcine oocyte system could help to provide some novel insights of DMSO usage on female reproduction in humans and farm animals.

## Supporting Information

S1 Fig Cumulus expansion of porcine COCs treated with DMSO at different concentrations. (TIF)

S2 Fig Different types of chromatin abnormality observed in 4% DMSO treated porcine oocytes at 44h of IVM. (TIF)

S1 Table Primers used for real-time PCR. (DOC)

S2 Table Number distribution of oocytes with different types of chromatin abnormality in 4% DMSO treatment groups. (DOC)
