# Supplementary figures and images for: Dimethyl Sulfoxide Perturbs Cell Cycle Progression and Spindle Organization in Porcine Meiotic Oocytes
Source: PLoS One. 2016 Jun 27;11(6):e0158074. doi: 10.1371/journal.pone.0158074 (PMC4922549; doi:10.1371/journal.pone.0158074)

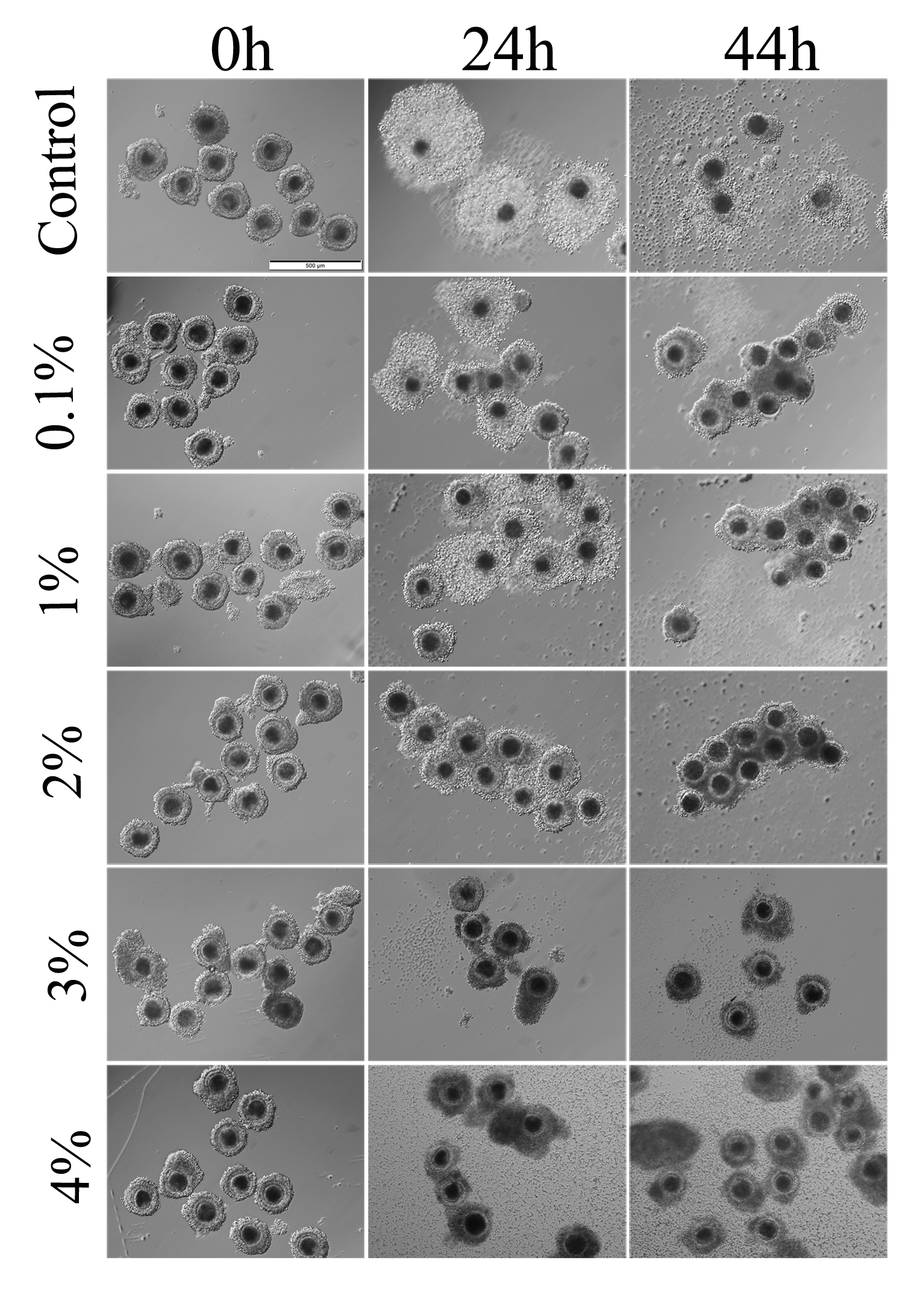

Supplement: S1 Fig — (TIF) [file pone.0158074.s001.tif]

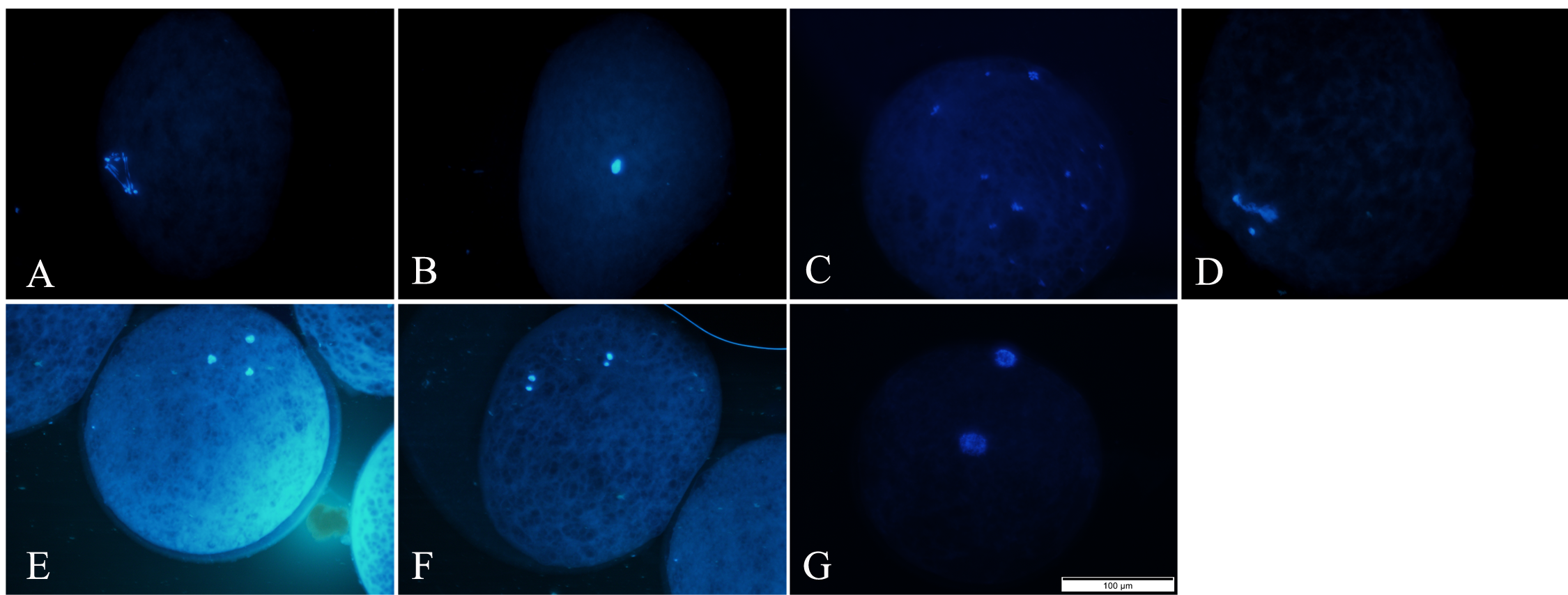

Supplement: S2 Fig — (TIF) [file pone.0158074.s002.tif]
