# Supplementary material for: Dimethyl Sulfoxide Perturbs Cell Cycle Progression and Spindle Organization in Porcine Meiotic Oocytes
Source: PLoS One. 2016 Jun 27;11(6):e0158074. doi: 10.1371/journal.pone.0158074 (PMC4922549; doi:10.1371/journal.pone.0158074)
Supplement: S1 Table — (DOC) [file pone.0158074.s003.doc]

| Genes | Gene Bank accession No. | Primer sequence(5’-3’) | Tm  (℃) | Product size(bp) |
| --- | --- | --- | --- | --- |
| Oct4 | NM_001113060.1 | F: GGGGGTTCTCTTTGGGAAGG | 60 | 128 |
|  |  | R: TGTTGTCAGCTTCCTCCACC |  |  |
| Sox2 | NM_001123197.1 | F: CCCGTGGTTACCTCTTCTTCC | 60 | 172 |
|  |  | R: CGTTGATGGCCGTGCC |  |  |
| Lin28 | NM_001123133.1 | F: GTTCGGCTTCCTGTCCATGA | 60 | 125 |
|  |  | R: CCACAGCCTCACCCTCCTT |  |  |
| Mad2 | XM_003129223.3 | F: ATGACAGTGCACCCAGAGAA | 60 | 182 |
|  |  | R: TGGTCCCGATTCTTCCCATT |  |  |
| Bub1 | XM_013995819.1 | F: TCAAAGGGCAGGTTGCAGAA | 60 | 134 |
|  |  | R: TGACTTCACTCGGAACACCG |  |  |
| NF-κB | NM_001048232.1 | F: CTGAGTGCTGCTCCTTCCAA | 60 | 234 |
|  |  | R: CCGGAGCTCGTCTATTTGCT |  |  |
| Pten | NM_001143696.1 | F: AAAGCTGGAAAGGGACGAA | 60 | 147 |
|  |  | R: GCGCCTCTGACTGGGAAT |  |  |
| Bcl2 | XM_003121700.4 | F: CGTCCCAGCTCCACATCACC | 60 | 130 |
|  |  | R: AGTGCCCCACCGAAGGAGAA |  |  |
| Caspase3 | NM_214131.1 | F: TTTGCGTGCTTCTAAGCCAT | 60 | 147 |
|  |  | R: GGCAGGCCTGAATTATGAAA |  |  |
| Caspase9 | XM_013998997.1 | F: CCAGTCTGCCCACACCTAGT | 60 | 175 |
|  |  | R: ACAGCATTGGAGACCCTGAG |  |  |
| Ywhag | XM_003124396.4 | F: GGCCATGAAGAACGTGACAG | 60 | 224 |
|  |  | R: CATCCTGACATACGGCCTCC |  |  |

**S1 Table. Primers used for real-time PCR.**
