# Supplementary material for: Dimethyl Sulfoxide Perturbs Cell Cycle Progression and Spindle Organization in Porcine Meiotic Oocytes
Source: PLoS One. 2016 Jun 27;11(6):e0158074. doi: 10.1371/journal.pone.0158074 (PMC4922549; doi:10.1371/journal.pone.0158074)
Supplement: S2 Table — (DOC) [file pone.0158074.s004.doc]

**S2 Table. Number distribution of oocytes with different types of chromatin abnormality in 4% DMSO treatment groups.**

|  | A | B | C | D | E | F | G | Total |
| --- | --- | --- | --- | --- | --- | --- | --- | --- |
| 24h DMSO + 20h TCM-199 | 0 | 7 | 8 | 1 | 5 | 4 | 2 | 27 |
| 24h DMSO + 20h DMSO | 8 | 8 | 7 | 1 | 5 | 2 | 1 | 32 |
